# Do the current MS clinical course descriptors need to change and if so how? A survey of the MS community

**DOI:** 10.1177/13524585231196786

**Published:** 2023-09-11

**Authors:** Alan J Thompson, Marcello Moccia, Maria Pia Amato, Peter A Calabresi, Marcia Finlayson, Annie Hawton, Fred D Lublin, Ruth Ann Marrie, Xavier Montalban, Michael Panzara, Maria Pia Sormani, Jon Strum, Barbara G Vickrey, Timothy Coetzee

**Affiliations:** Queen Square Multiple Sclerosis Centre, Department of Neuroinflammation, UCL Queen Square Institute of Neurology, NIHR University College London Hospitals Biomedical Research Centre, Faculty of Brain Sciences, University College London, London, UK; Department of Molecular Biology and Molecular Biotechnology, Federico II University of Naples, Naples, Italy Multiple Sclerosis Unit, Policlinico Federico II University Hospital, Naples, Italy; Department NEUROFARBA, Section of Neurosciences, University of Florence, Florence, Italy IRCCS Fondazione Don Carlo Gnocchi, Florence, Italy; Department of Neurology and The Solomon H. Snyder Department of Neuroscience, Johns Hopkins University School of Medicine, Baltimore, MD, USA; School of Rehabilitation Therapy, Queens University, Kingston, ON, Canada; University of Exeter Medical School, University of Exeter, Exeter, UK; Department of Neurology, Icahn School of Medicine at Mount Sinai, New York, NY, USA; Departments of Medicine & Community Health Sciences, Max Rady College of Medicine, Rady Faculty of Health Sciences, University of Manitoba, Winnipeg, MB, Canada; Multiple Sclerosis Centre of Catalonia and Department of Neurology-Neuroimmunology, Hospital Universitari Vall d’Hebron, Universitat Autònoma de Barcelona, Barcelona, Spain; Neurvati Neurosciences, New York, NY, USA; Department of Health Sciences, University of Genoa, Genoa, Italy IRCCS Ospedale Policlinico San Martino, Genoa, Italy; RealTalk MS, Long Beach, CA, USA; Department of Neurology, Icahn School of Medicine at Mount Sinai, New York, NY, USA; National Multiple Sclerosis Society, 733 Third Avenue, New York, NY 10017, USA; National Multiple Sclerosis Society, New York, NY, USA

**Keywords:** Multiple sclerosis, progression, relapses, clinical course, progressive multiple sclerosis, relapsing multiple sclerosis

## Abstract

**Background and Objectives:**

The current clinical course descriptors of multiple sclerosis (MS) include a combination of clinical and magnetic resonance imaging (MRI) features. Recently there has been a growing call to base these descriptors more firmly on biological mechanisms. We investigated the implications of proposing a new mechanism-driven framework for describing MS.

**Methods:**

In a web-based survey, multiple stakeholders rated the need to change current MS clinical course descriptors, the definitions of disease course and their value in clinical practice and related topics.

**Results:**

We received 502 responses across 49 countries. In all, 77% of the survey respondents supported changing the current MS clinical course descriptors. They preferred a framework that informs treatment decisions, aids the design and conduct of clinical trials, allows patients to understand their disease, and links disease mechanisms and clinical expression of disease. Clinical validation before dissemination and ease of communication to patients were rated as the most important aspects to consider when developing any new framework for describing MS.

**Conclusion:**

A majority of MS stakeholders agreed that the current MS clinical course descriptors need to change. Any change process will need to engage a wide range of affected stakeholders and be guided by foundational principles.

## Introduction

Since the publication of the Lublin–Reingold clinical course descriptors for multiple sclerosis (MS) in 1996, and the revised descriptors in 2013, there have been calls for the development of course descriptors anchored to the biological mechanisms of the disease.^1^

Some conceptual models have been suggested, but as yet none have been widely adopted (Antel, Antel and Caramanos, 2012).^2,3,4^ Recently, the International Advisory Committee on Clinical Trials in Multiple Sclerosis (an international group of experts convened under the auspices of the European Committee for Treatment and Research in MS and the US National Multiple Sclerosis Society) revisited the current clinical course descriptors, beginning with an exploration of how recent developments in our understanding of disease progression should be integrated to the existing paradigm. This effort culminated in the publication of a proposal for a new mechanism-driven framework of MS progression. The proposed framework posits that long-term disability progression results from a combination of pathological processes, which vary between individuals and within individuals over time and with ageing.^5^ Disability progression can occur from the early phases of relapsing MS, is partially independent of relapse activity, and is associated with concomitant accelerated brain, spinal cord, and retinal atrophy, ultimately leading to worse long-term outcomes.^6–11^ As such, the challenge is using clinician assessed and performance measures, patient reported outcomes and biomarkers to identify the wide range of MS pathology mechanisms, from disease onset and through its course, and related clinical expression.^2,5^ The emergence of treatment options with proven efficacy in progressive disease has further highlighted the need to identify and tackle biologic mechanisms of MS progression at the individual level.^12^

The recognition of shared underlying biology between relapsing and progressive MS blurs the distinction between these forms of the disease as expressed in the current clinical course descriptors, raising important questions about how the descriptors should evolve or whether they should be replaced with a new approach. Consequently, a working group convened under the auspices of the International Advisory Committee on Clinical Trials in MS is building on the proposed progression framework and developing a new mechanism-driven model – and an associated implementation roadmap – for describing MS. Since new descriptors of MS can potentially affect a wide range of stakeholders, including people with MS (i.e. informed decision-making), clinicians (i.e. prognosis, treatment), researchers (i.e. link between disease mechanisms and clinical expression), industry (i.e. design and conduct of clinical trials), regulators and policymakers (i.e. drug approval and access), it is essential to understand the implications of their adoption. In this paper, we report on a landscape assessment conducted to understand community perspectives on the need to change the MS clinical course descriptors and expectations for a new approach to describing MS.

## Method

### Study design

This was a cross-sectional study using a structured web-based questionnaire designed and reported following the Consensus-Based Checklist for Reporting of Survey Studies (CROSS).^13^ It was developed by an *ad hoc* working group of the International Advisory Committee on Clinical Trials in MS – a global body sponsored by the European Committee for Treatments and Research in MS (ECTRIMS) and the National Multiple Sclerosis Society (USA). The Committee has been in existence for over 30 years and is composed of experts in clinical trials and clinical research in MS. The committee is charged by the sponsoring organizations with providing perspective and guidance to the scientific and clinical community related to planning and implementation of clinical trials of MS therapies and allied topics. The current membership of the committee can be accessed here: – https://www.ectrims.eu/wpcontent/uploads/2023/03/Clinical-Trials-Cmte-Roster-2023-24.pdf.

The web-based questionnaire was built upon the Survey Monkey® software platform (https://www.surveymonkey.com/). A consent statement was provided, and completion of the survey was taken to imply consent. All participants consented to the questionnaire and the presentation of data at an aggregate level.

#### Survey design

The survey was designed to gather perspectives on current MS clinical course descriptors and future frameworks, potentially including laboratory and imaging biomarkers of different injury and compensatory mechanisms. Overall, the survey consisted of four sections, with 53 close-ended questions, and 2 open-ended questions. In section 1 (6 questions), participants were asked about their level of agreement with the statement ‘the current MS clinical course descriptors need to change’ (numeric slider scale from 0 = disagree completely, 50 = neutral to 100 = agree completely) and their views on the current MS clinical course descriptors (5-point Likert-type scale with strongly disagree, disagree, neither agree or disagree, agree, strongly agree). Section 2 asked participants for their perspectives on a future framework (8 questions) (5-point Likert-type scale with not at all important, neutral, somewhat important, very important, extremely important), the importance of monitoring different injury and compensatory mechanisms in a new MS progression framework (8 questions) (Likert-type scale with not at all important, neutral, somewhat important, very important, extremely important), and the readiness of laboratory and imaging biomarkers for use in disease management at the individual level (20 questions) (4-point Likert-type scale: unsure, unlikely to be useful, needs additional research, ready for use). In section 3, participants rated the importance of different aspects of a new framework for MS progression (6 questions) (Likert-type scale with not at all important, neutral, somewhat important, very important, extremely important). Two open-ended questions were also made available to discuss implications for patients that the working group should consider, along with any other perspectives or comments. Finally, section 4 (5 questions) collected the role in the MS community, category of healthcare professional, gender identity, age range (18–24, 25–34, 35–44, 45–54, 55–64, >65) and country of residence (which we grouped into main continents: Africa/Middle East, Asia/Oceania, Europe, South America and North America). The survey is presented in the Supplemental Material.

#### Survey pretesting and dissemination

The survey instrument was developed in English, and iteratively tested by a subset of the author group from different countries (A.J. Thompson, M. Moccia, P.A. Calabresi, F.D. Lublin, R.A. Marrie, M. Panzara, and T. Coetzee). Through two rounds of review the instrument was refined to reduce the number of survey questions, to favour question formats using Likert-type responses, and to limit the number of responses with open text.

We sought to obtain input from a broad range of stakeholders, including healthcare professionals, researchers, people with MS, organization representatives, policymakers and pharmaceutical industry representatives from around the world. Therefore, we distributed the survey to multiple organizations across several continents, including the executive committees of ECTRIMS, the Americas Committee for Treatments and Research in MS (ACTRIMS), the Latin American Committee for Treatment and Research in MS (LACTRIMS) and the Middle North Africa Committee for Treatments and Research in MS (MENACTRIMS).

Additional invitations to participate in the survey were distributed by email to the Canadian Network of MS Clinics, clinics affiliated with the Italian MS Register as well as investigators associated with the Magnetic Resonance Imaging in MS (MAGNIMS) network and the North American Imaging in MS Cooperative (NAIMS). In addition, email invitations were distributed to the wider MS research and health professional community registered with the NMSS, the Australian, Italian and UK MS Societies, as well as the Consortium of MS Centres (North America). To capture individuals who did not identify with any specific professional organization or network, the survey was also distributed via social media (Linkedin, Twitter). Specifically, the survey was disseminated once via email to the professional organizations and posted once on social media. The survey was opened on 12 December 2022 and closed on 26 January 2023; interim monitoring of respondents was performed to evaluate the representativeness of the sample. Owing to the descriptive nature of the study, we did not set sample size *a priori*.

### Statistical analyses

Data were extracted from Survey Monkey (https://www.surveymonkey.com/) and processed using Microsoft Excel. We planned *a priori* to exclude individual participants with more than 20% missing responses to be excluded from the analyses, to avoid bias from survey participants with limited/focused knowledge on investigated topics or that might have mistakenly submitted incomplete questions.^14^ We note that there is no mechanism for assessing the response rate, nor for detecting and eliminating duplicate survey submissions.

Descriptive results are presented as mean (standard deviation), number (percent) or median (range), as appropriate. Based on our aim (having a landscape assessment), we specifically decided not to use statistical tests to evaluate associations between the main variables of interest (need to change the MS clinical course descriptors and expectations for a new approach to describing MS) and other variables (e.g. demographics) that would have focused the attention on the perspectives of a subgroup of the community.

### Data availability statement

The datasets generated during and/or analysed during the current study are available from the corresponding author on reasonable request.

## Results

### Characteristics of survey participants

Over a 45-day period from December 2022 to January 2023, we received 502 responses across 49 countries. The completeness to individual questions ranged from 92.7% in more general questions (e.g. attitudes towards the current clinical course descriptors), to 77.7% in more technical questions (e.g. readiness of imaging measures). None of the participants had more than 20% responses as missing. Most respondents (86.8%) were healthcare professionals and researchers. Among the healthcare professionals, most respondents were MS neurologists, followed by general neurologists and MS nursing professionals. The full representation of gender, age, countries and roles in the MS community and healthcare services is presented in Table 1.

**Table 1.** Demographic and other characteristics of survey respondents.^a^

Questions	Answers	Percent
What is your gender identity? (*N* = 383)		
	Female	40.2
	Male	55.3
	Other/neither/prefer not to say	4.5
What is your age? (*N* = 381)		
	18–34	7.1
	35–44	24.4
	45–54	28.1
	55–64	24.7
	>65	15.7
In what country do you live? (*N* = 334)		
	Africa/Middle East	6.5
	Asia/Oceania	6.7
	Europe	24.8
	Latin America	14.7
	North America	47.3
Please indicate your primary role in the MS community. (*N* = 386)		
	Healthcare professional	67.6
	Researcher	19.2
	Person affected by MS (patient, caregiver, family member, etc.)	5.2
	Other	3.9
	Patient organization representative	2.3
	Pharmaceutical industry professional	1.3
	Policymaker (e.g. FDA or EMA official, health insurance industry professional, etc.)	0.5
If ‘Healthcare professional’ was selected, what category best describes you as a health professional. (*N* = 259)		
	MS neurologist	73.7
	General neurologist	13.1
	MS nurse or nurse practitioner	4.8
	Other	2.3
	Trainee neurologist	1.6
	Physical therapist	1.3
	Psychologist or psychiatrist	0.8
	Occupational therapist	0.8
	Pharmacist	0.8
	Physiatrist	0.4

a Missingness ranged from 23.1% to 33.5% across the first four questions.

### Perspectives on the current clinical course descriptors

**Figure 1.**
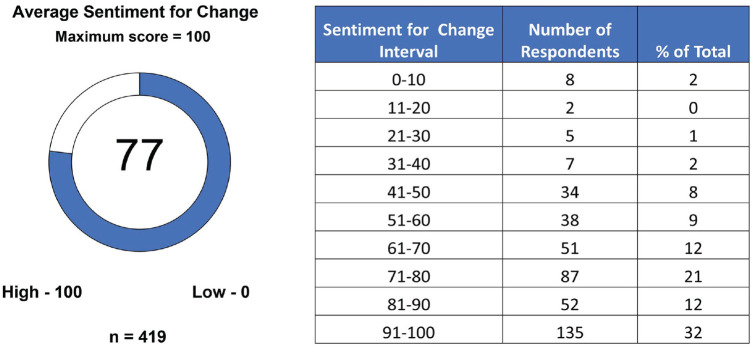
Agreement for changing the current clinical course descriptors. Participants expressed their agreement for change in the clinical course descriptors on a scale of 0–100. The average sentiment (left) is indicated. The number of responses by 10-point increments and the percentage of the total are shown on the right.

There was overall agreement that the current MS clinical course descriptors need to change (77 ± 22 average rate of agreement) (Figure 1). Two-thirds indicated a level of agreement for change between 71 and 100.

**Figure 2.**
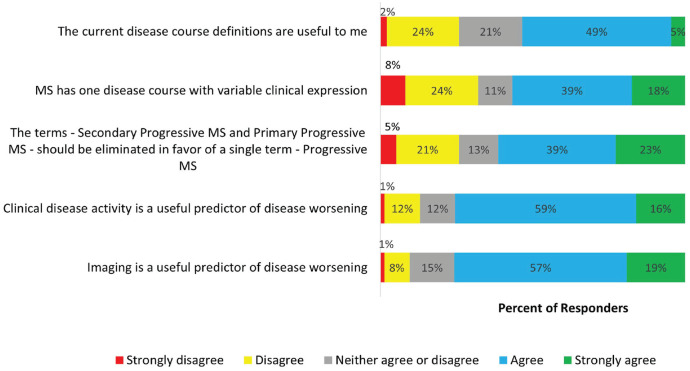
Attitudes towards the current clinical course descriptors. Participants were offered several statements related to the current clinical course descriptors and related topics. The stacked bars reflect the level of agreement or disagreement with the statement.

Respondents expressed varying perspectives on the current clinical course descriptors. A total of 53% found the current course descriptors useful, while the remainder were neutral or disagreed with this statement. A majority (62%) favoured elimination of the terms Secondary Progressive MS and Primary Progressive MS in favour of a single term – progressive MS. The highest rate of agreement (agree and strongly agree) was with the following statements: imaging is a useful predictor of disease worsening (75.6%) and clinical disease activity is a useful predictor of disease worsening (75.5%) (Figure 2).

### Perspectives on a future framework for describing MS

**Figure 3.**
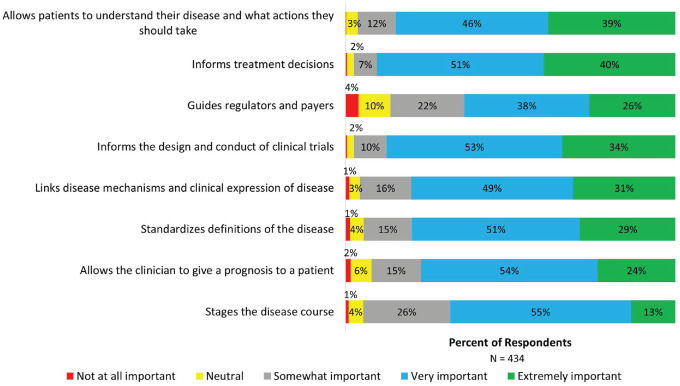
Considerations for a new framework for describing MS. Stacked bars show the percentage of respondents rating the importance of different aspects of a new framework for describing MS.

In considering a new framework for describing MS, respondents placed the highest importance (very important and extremely important) on its role in informing treatment decisions (90.4%), guiding the design and conduct of clinical trials (87.5%), allowing patients to understand their disease and what actions they should take (84.7%), and linking disease mechanisms and clinical expression of disease (80.1%) (Figure 3).

**Figure 4.**
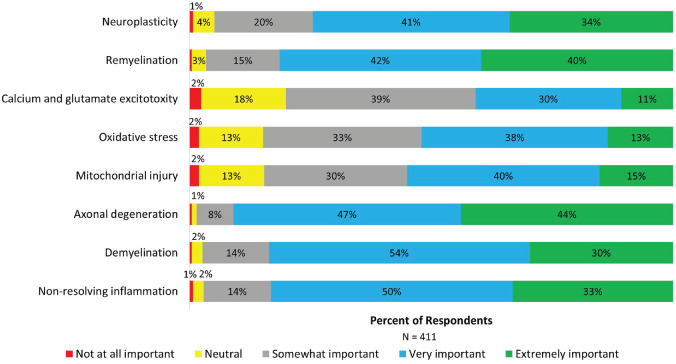
Perspectives on disease mechanisms to be measured by a new disease course framework. Stacked bars show the percentage of respondents rating the importance of different disease mechanisms to be measured by a new framework for describing MS.

Disease mechanisms that were reported as having the highest importance (very important and extremely important) for measurement in a new disease course framework included axonal degeneration (90.9%), demyelination (83.6%), non-resolving inflammation (83.2%) and remyelination (81.4%). Fewer than 50% of respondents considered disease mechanisms of oxidative stress and calcium and glutamate excitotoxicity as having high importance (Figure 4).

In considering the tools and measures that could be employed to assess disease mechanisms in a new disease course framework, respondents considered magnetic resonance imaging (MRI) for lesion volume and count (83.2%), cerebrospinal fluid (CSF) oligoclonal bands (61.9%) and MRI for global and regional brain atrophy (61.2%) to have the highest readiness for use. A range of other measures and tools (e.g. serum nerve fibre layer (NfL), genetic tests, MRI for central vein sign, etc.) were not considered ready for use and required more research (Table 2).

**Table 2.** Measures and tools for a new framework for describing MS.^a^

Which measures and tools are ready for use in disease management in an individual patient?	Unsure (%)	Unlikely to be useful (%)	Needs additional research (%)	Ready for use (%)
Biological measures (*N* = 413)				
Pathological specimens	11.0	23.9	58.0	7.1
Genetic markers of progression	7.3	14.6	74.5	3.6
Serum and CSF biomarkers (*N* = 394)				
Serum neurofilament light chain	5.9	7.6	49.5	37.0
Serum GFAP	9.8	8.5	71.9	9.8
CSF – oligoclonal bands	5.4	20.9	11.8	61.9
CSF – IgG Index	7.0	23.8	17.6	51.6
CSF – Kappa free light chains	12.0	17.9	44.4	25.7
CSF NfL	7.2	16.7	49.9	26.2
CSF GFAP	11.8	17.0	61.4	9.8
Imaging measures (*N* = 390)				
MRI for lesion volume and count	25.6	3.1	11.1	83.2
MRI for central vein sign	6.0	14.0	35.1	44.9
MRI for paramagnetic (iron) rim lesions	6.5	4.6	53.2	35.7
MRI for intralesional axonal loss	9.4	4.9	68.1	17.6
MRI for global and regional brain atrophy	3.7	4.9	30.2	61.2
MRI for spinal cord atrophy	3.1	3.9	41.3	51.7
Magnetic resonance spectroscopy	10.2	23.2	52.6	14.0
PET	12.2	21.5	56.7	9.6
Functional MRI	10.2	25.5	50.5	13.8
Optical coherence tomography	6.0	6.0	30.8	57.2
Visual evoked potentials	5.5	25.0	16.1	53.4

CSF: cerebrospinal fluid; NFL: nerve fibre layer; MRI: magnetic resonance imaging; PET: positron emission tomography.

a The percentage of respondents rating the readiness of measures and tools for assessing disease mechanisms in a new framework for describing MS.

**Figure 5.**
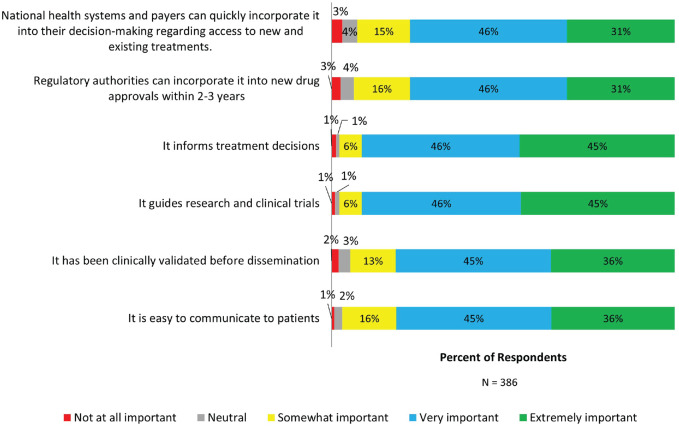
Expectations of a new disease course framework for MS. Stacked bars show the percentage of respondents rating the importance of different dimensions of a new framework for describing MS.

Respondents were also asked to rate their expectations for the performance of a new framework. Highest importance (very important and extremely important) was placed on the framework’s ability to inform treatment decisions (91%), guide research and clinical trials (91%), and ease of communication to patients (81%). Most (81%) expected that a new framework should be clinically validated before dissemination and also adopted by regulatory authorities (77%) and national health systems and payers (77%) (Figure 5).

## Discussion

The emergence of clinical course descriptors for MS resulted in a consistent approach to patient selection for clinical trials.^15^ This reduction in patient heterogeneity presaged nearly three decades of successful clinical trials and a transformation in the clinical management of relapsing MS. Along the way, the course descriptors were widely integrated in the MS healthcare ecosystem. People living with MS rely on them to understand their status in the disease course, and clinicians employ the descriptors in the management of the disease. They are central to defining the patient populations for contemporary clinical trials and clinical studies. Moreover, regulatory authorities and health technology assessment agencies have incorporated the descriptors in drug approvals and reimbursement schemes.^5^

Yet, despite widespread use, these survey results indicate community support to consider replacement of the existing clinical course descriptors with a mechanism-driven framework. The survey results also illustrate the complexity that will accompany introduction of a new approach to describing MS. While most respondents supported changing the current clinical course descriptors, the survey results highlight community expectations that a new framework reflects the current understanding of biological mechanisms of MS, with domain-based staging (e.g. focal inflammatory lesions, degree of axonal injury, microglial activation, etc.) that links disease mechanisms to a clinical descriptor. Furthermore, there is also an expectation that a new framework has adequate clinical validation before introduction.

While adopting a mechanism-based approach to describing MS is attractive, we must also recognize some of the limitations with current tools to assess disease mechanisms within individual patients. Our survey findings demonstrated that participants only expressed confidence in MRI for lesion volume and count, CSF oligoclonal bands, and MRI for global and regional brain atrophy as tools for disease management. Laboratory and MRI advances might soon allow the application in clinical practice of tools in a range of areas – serum/CSF NfL, serum/CSF GFAP, CSF kappa free light chains, MRI for central vein sign, MRI for paramagnetic (iron) rim lesions, MRI for intralesional axonal loss, MRI for spinal cord atrophy, optical coherence tomography for retinal NFL (axons) and ganglion cell layer (neurons) thinning, and visual evoked potentials – though these remain in development and may not be equally applicable to the framework.^16–23^ Hence, a new framework will need to be sufficiently flexible to allow for timely incorporation of new tools and measures as clinically validated tools become available. It is reasonable to consider the development of an initial minimum toolkit for diagnosis and prognostication, to which new measures or tools are integrated based on the accumulating evidence over time. Concerted effort with multiple stakeholders will be required to ensure progress in the development and implementation of requisite measurement tools.

The wide use of current clinical course descriptors poses considerable challenges to future efforts to introduce a new approach to describing MS. Attention will need to be paid to engaging all the affected stakeholders in developing and adopting a change process following introduction of a new approach to describing MS. In addition, the stakeholders will need to be engaged in the development of strategies to clinically validate the proposed framework. Concerted efforts by researchers, clinicians, patients and funders will be essential to success.

The working group leading this initiative proposes six principles to guide the development of a new approach for describing MS. First, a new framework should reflect the currently understood domains of biological mechanisms of disease. Second, it must acknowledge that knowledge gaps exist and plan to address them over time, as new information emerges.

Third, it should drive and stimulate advances in knowledge and therapeutic innovations.

Fourth, it will need to be easily understood and facilitate communication with patients. Fifth, the principles should have equity at the core of their development and application so that it will be feasible to adopt the framework globally. Sixth, the framework should be meaningful, useful and applicable to all stakeholders.

A related consideration is the link between diagnosis and subsequent disease course. For more than two decades, the diagnosis of MS has been guided by the McDonald diagnostic criteria.^24^ However, the diagnosis of MS has yet to be meaningfully connected to the course of the disease. While the 2017 edition of the McDonald Criteria took steps in this direction, the introduction of a new framework poses opportunities to provide a closer interconnection.^25^ Moreover, the expansion of the MS disease course spectrum to include Clinically Isolated Syndrome and Radiologically Isolated Syndrome emphasizes the need to link diagnosis and disease course description – both for patient management and for guiding research.^25,26^ Such an effort – while challenging – has the potential to simplify both diagnosis and prognosis.

A shift from strict labels towards characterizing how biological disease is connected to clinical presentation would mark an important advance in current practice – and one with precedent in other neurological diseases. Mechanism-driven frameworks for disease staging paradigms are now guiding clinical trials in Huntington disease and Alzheimer disease, and, soon, neuronal alpha-synuclein diseases.^27–29^ The application of a similar mechanism-driven framework to MS could potentially address biologic and pathologic heterogeneity in patients at variable stages of disease and ages, and offer promise to treat early and ideally ahead of irreversible deficits. It is reasonable to expect that it would also improve current clinical practice and contribute to development of precision medicine approaches to MS.

### Limitations

There are several limitations to our study. These include the possibility of selection bias, which is likely in Internet-based questionnaires. However, in our study, we found good completeness of response to questions (excluding demographic questions) ranging from 77% to 93%. Our population had reasonable representation between age ranges and genders. However, looking at the worldwide distribution, our sample over-represented North America, Europe and South America, and only included a minority of responses from Africa/Middle East and Asia/Oceania, potentially limiting generalizability. As such, additional efforts will need to ensure that perspectives from countries underrepresented in this survey are fully reflected in new MS descriptors, including offering the survey in multiple languages, distributing the survey multiple times and providing longer windows for survey responses. Global, representative input is especially important due to the different distribution in relapsing and progressive forms of MS (and, possibly, of underlying mechanisms) between different racial and ethnic groups.^30^,^31^

Also, the survey population was largely healthcare professionals (i.e. MS neurologists) and researchers, and only included small numbers of people with MS and their organizations’ representatives. While we believe our sample is fit for purpose, further efforts will be needed to incorporate perspectives and experiences from people with MS. Our survey did not focus on clinical measures, which are very likely to miss subtle but meaningful clinical events. However, in the future, digital biomarkers, wearables and smartphone apps might improve the sensitivity to change in clinical practice, and may inform clinical determination of the course descriptors.

## Conclusion

The findings of this survey support the ongoing international effort to revisit the current clinical course descriptors for MS. It has also provided insights to guide an implementation roadmap for a new mechanism-driven framework. Future challenges include designing the optimum approach for defining and describing MS across the continuum of the disease course. Engaging key informants from a range of perspectives (e.g. industry, general neurologists, patients, policymakers) in future work will ensure that we can capture nuanced feedback. Ultimately, a robust dissemination and engagement strategy will need to be developed to ensure that any proposed framework is validated and refined through research studies, and widely adopted by affected stakeholders.

## Supplemental Material

sj-pdf-1-msj-10.1177_13524585231196786 – Supplemental material for Do the current MS clinical course descriptors need to change and if so how? A survey of the MS community Click here for additional data file. Supplemental material, sj-pdf-1-msj-10.1177_13524585231196786 for Do the current MS clinical course descriptors need to change and if so how? A survey of the MS community by Alan J Thompson, Marcello Moccia, Maria Pia Amato, Peter A Calabresi, Marcia Finlayson, Annie Hawton, Fred D Lublin, Ruth Ann Marrie, Xavier Montalban, Michael Panzara, Maria Pia Sormani, Jon Strum, Barbara G Vickrey and Timothy Coetzee in Multiple Sclerosis Journal
