# Supplementary material for: Do the current MS clinical course descriptors need to change and if so how? A survey of the MS community
Source: Mult Scler. 2023 Sep 11;29(11-12):1363–72. doi: 10.1177/13524585231196786 (PMC10580678; doi:10.1177/13524585231196786)
Supplement: sj-pdf-1-msj-10.1177_13524585231196786 – Supplemental material for Do the current MS clinical course descriptors need to change and if so how? A survey of the MS community [file sj-pdf-1-msj-10.1177_13524585231196786.pdf]

National Multiple Sclerosis Society

ECTRIMS
EUROPEAN COMMITTEE FOR TREATMENT AND RESEARCH IN MULTIPLE SCLEROSIS

International Advisory Committee on Clinical Trials in MS

Overview

Since the introduction of the Lublin-Reingold clinical course descriptors for MS there have been calls for the development of a disease classification rooted in the biological mechanisms of the disease. The International Advisory Committee on Clinical Trials in MS has begun to address these calls with a publication proposing a new mechanism-driven framework toward defining MS progression.

A working group convened by the committee is creating a roadmap for the development and implementation of a mechanism-driven description of the disease course of MS proposed in the committee's recent publication (Kuhlmann et al., Lancet Neurology, 2022)

Thank you for taking the time to share your perspectives on this important topic. Your responses to this survey will inform our efforts to chart a new path for how we describe MS.

Informed consent

By completing this survey, you agree to the use of your responses will inform the work of the International Advisory Committee on Clinical Trials in MS.

1. If you would like to get updates or contribute to future surveys, please enter your email address.

Your Perspectives on the Current Clinical Course Descriptors for MS

2. Please indicate your level of agreement with the following statement:

The current MS clinical course descriptors need to change.

Disagree completely — Neutral — Agree completely

3. Please indicate your agreement or disagreement with the following statements.

	Strongly disagree	Disagree	Neither agree or disagree	Agree	Strongly agree
The current disease course definitions are useful to me	○	○	○	○	○
MS has one disease course with variable clinical expression	○	○	○	○	○
The terms - Secondary Progressive MS and Primary Progressive MS - should be eliminated in favor of a single term - Progressive MS	○	○	○	○	○
Clinical disease activity is a useful predictor of disease worsening	○	○	○	○	○
Imaging is a useful predictor of disease worsening	○	○	○	○	○

Your Perspectives on a Future Framework of MS Progression

Kuhlmann et al. propose the development of a new framework for MS progression to guide research and patient care. In this section, we ask for your perspectives on different aspects of a potential new framework.

4. Please rate the importance of different aspects of a new framework for MS progression.

	Not at all important	Neutral	Somewhat important	Very important	Extremely important
Stages the disease course	○	○	○	○	○
Allows the clinician to give a prognosis to a patient	○	○	○	○	○
Standardizes definitions of the disease	○	○	○	○	○
Links disease mechanisms and clinical expression of disease	○	○	○	○	○
Informs the design and conduct of clinical trials	○	○	○	○	○
Guides regulators and payers	○	○	○	○	○
Informs treatment decisions	○	○	○	○	○
Allows patients to understand their disease and what actions they should take	○	○	○	○	○

Your Perspectives on a Future Framework of MS Progression

5. Please rate the importance of monitoring the following injury and compensatory mechanisms in a new MS progression framework. Please provide your perspective whether or not tests are available today or still need to be developed to monitor a pathway.

	Not at all important	Neutral	Somewhat important	Very important	Extremely important
Non-resolving inflammation	◯	◯	◯	◯	◯
Demyelination	◯	◯	◯	◯	◯
Axonal degeneration	◯	◯	◯	◯	◯
Mitochondrial injury	◯	◯	◯	◯	◯
Oxidative stress	◯	◯	◯	◯	◯
Calcium and glutamate excitotoxity	◯	◯	◯	◯	◯
Remyelination	◯	◯	◯	◯	◯
Neuroplasticity	◯	◯	◯	◯	◯

6. Which of the following biological measures are ready for use in disease management (not diagnosis) in an individual patient?

	Unsure	Unlikely to be useful	Needs additional research	Ready for use
Pathological specimens	◯	◯	◯	◯
Genetic markers of progression	◯	◯	◯	◯

Your Perspectives on a Future Framework of MS Progression

7. Which of the following serum or cerebrospinal (CSF) biomarkers are ready for use in disease management (not diagnosis) in an individual patient?

	Unsure	Unlikely to be useful	Needs additional research	Ready for use
serum Neurofilament light chain (NfL)	○	○	○	○
serum Glial Fibrillary Acidic Protein (GFAP)	○	○	○	○
CSF - Oligoclonal bands	○	○	○	○
CSF - IgG Index	○	○	○	○
CSF - Kappa free light chains	○	○	○	○
CSF - Neurofilament light chain (NfL)	○	○	○	○
CSF - Glial Fibrillary Acidic Protein (GFAP)	○	○	○	○

Other biomarkers that should be considered?

[ ]

8. Which imaging measures are ready for use in disease management (not diagnosis) in an individual patient?

	Unsure	Unlikely to be useful	Needs additional research	Ready for use
MRI for lesion volume and count	○	○	○	○
MRI for central vein sign	○	○	○	○
MRI for paramagnetic (iron) rim lesions	○	○	○	○
MRI for intralesional axonal loss	○	○	○	○
MRI for global and regional brain atrophy	○	○	○	○
MRI for spinal cord atrophy	○	○	○	○
Magnetic Resonance Spectroscopy	○	○	○	○
PET	○	○	○	○
Functional MRI	○	○	○	○
Optical Coherence Tomography	○	○	○	○
Visual evoked potentials	○	○	○	○

Additional imaging measures that should be considered?

Your Perspectives on a Future Framework of MS Progression

9. Please rate the importance of the following aspects of a new framework for MS progression.

	Not at all important	Neutral	Somewhat important	Very important	Extremely important
It is easy to communicate to patients	◯	◯	◯	◯	◯
It has been clinically validated before dissemination	◯	◯	◯	◯	◯
It guides research and clinical trials	◯	◯	◯	◯	◯
It informs treatment decisions	◯	◯	◯	◯	◯
Regulatory authorities can incorporate it into new drug approvals within 2-3 years	◯	◯	◯	◯	◯
National health systems and payers can quickly incorporate it into their decision-making regarding access to new and existing treatments.	◯	◯	◯	◯	◯

10. Are there implications for patients that the working group should consider? Please limit your response to two points.

11. Do you have any other thoughts or comments for the working group's consideration? Please limit your response to two points.

Demographic Information

12. Please indicate your primary role in the MS community.

- ◯ Person affected by MS (patient, caregiver, family member etc)
- ◯ Healthcare professional
- ◯ Researcher
- ◯ Policymaker (e.g. FDA or EMA official, health insurance industry professional etc)
- ◯ Pharmaceutical industry professional
- ◯ Patient organization representative
- ◯ Other (please specify)

[ ]

Health Professional Information

13. Please select the category that best describes you as a health professional.

- ◯ MS neurologist
- ◯ General neurologist
- ◯ Trainee neurologist
- ◯ MS Nurse or Nurse Practitioner
- ◯ MS Physician Assistant
- ◯ Psychologist or Psychiatrist
- ◯ Physical Therapist
- ◯ Physiatrist
- ◯ Occupational Therapist
- ◯ Pharmacist
- ◯ Other (please specify)

  [ ]

- ◯ Not a healthcare provider

14. What is your gender identity?

- ◯ Female
- ◯ Male
- ◯ Neither male nor female
- ◯ Prefer not to say
- ◯ Other (please specify)

15. What is your age?

- ◯ 18-24
- ◯ 25-34
- ◯ 35-44
- ◯ 45-54
- ◯ 55-64
- ◯ 65+

16. In what country do you live?
